# Two-Cloud-Servers-Assisted Secure Outsourcing Multiparty Computation

**DOI:** 10.1155/2014/413265

**Published:** 2014-05-28

**Authors:** Yi Sun, Qiaoyan Wen, Yudong Zhang, Hua Zhang, Zhengping Jin, Wenmin Li

**Affiliations:** ^1^State Key Laboratory of Networking and Switching Technology, Beijing University of Posts and Telecommunications, Beijing 100876, China; ^2^Brain Image Processing, Columbia University, New York, NY 10032, USA

## Abstract

We focus on how to securely outsource computation task to the cloud and propose a secure outsourcing multiparty computation protocol on lattice-based encrypted data in two-cloud-servers scenario. Our main idea is to transform the outsourced data respectively encrypted by different users' public keys to the ones that are encrypted by the same two private keys of the two assisted servers so that it is feasible to operate on the transformed ciphertexts to compute an encrypted result following the function to be computed. In order to keep the privacy of the result, the two servers cooperatively produce a custom-made result for each user that is authorized to get the result so that all authorized users can recover the desired result while other unauthorized ones including the two servers cannot. Compared with previous research, our protocol is completely noninteractive between any users, and both of the computation and the communication complexities of each user in our solution are independent of the computing function.

## 1. Introduction

Secure multiparty computation (SMC) [1–7] is dedicated to computing a certain function among a set of mutually distrusted participants on their private inputs without revealing private information. Informally speaking, assuming that there are *m* participants, *P*_1_, *P*_2_,…, *P*_*m*_, each of them has a private number, respectively, *x*_1_, *x*_2_,…, *x*_*m*_. They want to cooperate to compute the function *y* = *f*(*x*_1_, *x*_2_,…, *x*_*m*_) without revealing *x*_*i*_ of *P*_*i*_ to other parties *P*_*j*_, *j* ≠ *i*, *i*, *j* ∈ {1,…, *m*}, as well as guaranteeing that any unauthorized ones cannot get the result *y*. In the past, researchers mainly focused on designing the style of secure multiparty computation protocols by which users themselves cooperatively accomplish the function evaluation through their internal interactions [1, 3, 8–11]. The computation and communication complexities always depend polynomially on the complexity of the function to be computed. Therefore, users suffer from the heavy overload of these protocols.

The emergence of the cloud [12, 13] inspires users to apply the powerful computing ability of the cloud to help them to conduct complicated computations, that is, secure outsourcing computation [14–18] to the cloud. They expect that the cloud can independently complete any function computation on their outsourced data although the data has been encrypted by their own keys for security. Moreover, the final result should be kept private to the cloud even though it is the cloud that conducts all of the computations about the computing function. In this way, users only need to encrypt their data and decrypt the returned message to get the desired result. All computations about the computing function are in the charge of the cloud. There are no interactions between any users, and the computation and communication complexities of each user are independent of the computing function. However, this expectation is proven to be impossible in the single cloud server setting due to the impossibility of program obfuscation [19]. Therefore, in this paper, we try to realize it by introducing one more cloud server to the original model described above. More precisely, we consider the following scenario.

There are *m* + 2 distrusted parties including *m* users and two cloud servers in our system. We assume that all of them act semihonestly. The *m* users *P*_1_, *P*_2_,…, *P*_*m*_, with each having a private input, respectively, *x*_*i*_, as well as a pair of public-private keys (*pk*_*i*_, *sk*_*i*_), *i* = 1,…, *m*, encrypt their respective private inputs by their own public keys and then upload the ciphertexts of the inputs to a cloud server. They want to obtain the value *y* = *f*(*x*_1_, *x*_2_,…, *x*_*m*_) even if they may not be aware of what the computing function *f*(·) is by applying two cloud servers to operate on the outsourced encrypted data without revealing *x*_1_, *x*_2_,…, *x*_*m*_ and the result *y*.

In this paper, we study the outsourcing computation problem in multiple users-two-cloud-servers scenario and propose a two-cloud-servers-assisted secure outsourcing multiparty computation protocol to compute any function on lattice-based encrypted data under multiple keys of the users. Herein, we apply one cloud server called the storing-cloud (SC) to store the outsourced data encrypted by users and make a midtransformation to these ciphertexts once some function begins to be computed. We call the other cloud server the computing-cloud (CC). It is responsible for transforming the midtransformed ciphertexts by SC to the ones that are blinded by the same two private keys of the two assisted cloud servers so that CC can further compute *y* following the function on the ciphertexts. Finally, in order to protect the result, the two servers cooperatively produce a custom-made result for each user. Compared with previous solutions, our protocol has the following three advantages.

1. Our protocol is completely noninteractive between any users.
2. The cloud is to do all of the computations related to the computing function, while users would do nothing except for encrypting their private inputs and decrypting the returned result.
3. The computation and communication complexities of each user in our solution are independent of the computing function.

*Organization*. The rest of this paper is organized as follows. In Section 2, we briefly give an overview of some recent related works. Herein, we consider the problems in secure outsourcing computation from the point of view of the users and the cloud servers, respectively, and then rationally construct our protocol in the multiple users-two-cloud-servers setting. In Section 3, we briefly introduce a lattice-based encryption scheme and the security model and then present our protocol in Section 4 in detail. In Section 5, we analyze the proposed protocol in detail and give a strict proof based on real-ideal simulation paradigm. Finally, we summarize our work of this paper in the last section.

## 2. Related Works

According to previous research, there are many problems to be considered when outsourcing private data for function computation to the cloud. We discuss the difficulties in secure outsourcing computation to the cloud from the following two aspects.

*(1) To Users: Privacy of the Inputs and Results*. In secure outsourcing computation, users have to contribute their private data as the inputs of the function while not participating in the computation process. Moreover, all parties of the protocol including all users and cloud servers are mutually distrusted. Therefore, users would not like to submit their private data to the cloud. Allowing for security, a usual solution is to encrypt the private data before outsourcing them to the cloud. And there are some basic encryption models according to the encryption keys that users used.

In 2009, Gentry [20] presented a model where all users use a joint public key to encrypt their own private inputs while sharing the private key. Therein, the cloud cannot obtain the inputs or the result because they are protected by the encryption scheme, while the cloud does not have the private key. However, users have to participate in another interactive protocol to firstly recover the private key and then achieve the desired result. The processes, producing a joint public key, sharing the private key, and jointly recovering the result by their shared private key, bring large number of additional interactions among users, which is contrary to our expectation that we want to design a secure protocol with the least communications. Encrypting private data by the joint public key is not so satisfactory either. In cloud outsourcing scenario, it means that there are no interactions among the users whatsoever and the least two rounds of inevitable interactions between the user and the cloud server, sending out the inputs and receiving the result. Therefore, we look forward to a protocol with the least communications as well as low computations and high security. A recent work by Asharov et al. [21] proposes a scheme where users utilize their own public keys to encrypt their inputs, respectively, and guarantee that the cloud can succeed in computing the function on their private inputs by computing on the ciphertexts of the inputs encrypted under different keys. Although users still have to interact to obtain the result in the last step, encrypting respective input by the public key of each user is the best encryption model so far.

As to the privacy of the result, in 2011, Halevi et al. [22] proposed a noninteractive protocol to securely realize outsourcing computation. Therein, the server is entitled to learn the result. However, the computing result may be the vital information to the users in some scenario and so it cannot be revealed to others. Hence, besides the security of the inputs discussed above, users must consider the security of the result when constructing protocols. It should guarantee that any unauthorized users are not able to get the result although they may contribute their inputs and the cloud servers are not able to get the result although the result is computed by them. To this aspect, [20] has already protected the result by a joint public key of the users. However, this method is still not satisfactory since each authorized user is also not able to get it individually.

*(2) To Cloud Servers: Feasibility of Operating on Encrypted Inputs*. As discussed above, users would like to upload the encrypted inputs under their respective public keys to the cloud server rather than the original inputs. Therefore, the cloud servers, whose task is to compute a function on users' private inputs, would only obtain the ciphertexts of the inputs. That means that the cloud has to compute the function on users' private inputs through performing corresponding computations on the ciphertexts of the inputs encrypted by different public keys of users. As we know, fully homomorphic encryption (FHE) [20, 23] can operate on the ciphertexts of the inputs to compute the desired result produced by the inputs. But the usual FHE schemes are single-key schemes in the sense that they only can perform computations on ciphertexts encrypted under the same key. It is not feasible to conduct computations on the ciphertexts encrypted under different keys. In order to solve this problem, López-Alt et al. [24] propose a new FHE called multikey fully homomorphic encryption (MFHE) which has applied the techniques of bootstrapping, modulus reduction, and relinearization to operate on the ciphertexts of the inputs encrypted by multiple, unrelated keys. When outsourcing private data to the cloud, user can firstly encrypt it by its own key by applying MFHE. It is indeed the optimal solution from the point of view of the feasibility of ciphertexts and the privacy of inputs. However, as we mentioned before, it is still not satisfactory because users need to evaluate the decryption key and then use it to recover the result interactively by participating in another SMC protocol.

In fact, according to [19], it is proved that it is indeed impossible to construct a completely noninteractive protocol in the single server setting due to the impossibility of program obfuscation. Hence, if we want to obtain a secure protocol with complete noninteraction of users in outsourcing computation, we need at least two cloud servers.

In brief, allowing for the privacy of inputs and results from the perspective of users as well as the feasibility of operating on the outsourced encrypted data from the perspective of the cloud servers, if we want to construct a completely noninteractive secure outsourcing multiparty computation protocol where the computation and communication complexities of each user are independent of the computing function, we have the following conclusions.

1. All private data should be encrypted by the owners themselves using their respective public keys before outsourcing to the cloud servers.
2. The returned messages for each user should be different so that all authorized users can recover the final result by their respective private key but the unauthorized ones cannot.
3. It is reasonable to consider it in two-cloud-servers scenario.

## 3. Preliminaries

### 3.1. Lattice-Based Encryption

Since the privacy of the inputs and the computation complexity of each user depend on the encryption algorithm that the user used, an encryption scheme outstanding in both security and efficiency is the right one that users want to adopt. Hence, lattice-based encryption, which is against quantum attacks and is much more efficient than RSA and even the elliptic curve cryptosystem, becomes the first choice of rational users. Herein, we will show how the two cloud servers deal with the outsourced data encrypted by the lattice-based public key encryption scheme proposed in [24, 25] (denoted as LE scheme in this paper). Specifically, we recall it as follows.

*Notations*. Let *k* be the security parameter. Then, the LE scheme is parameterized by a prime *q* = *q*(*k*), a degree *n* polynomial *f*(*x*) ∈ *Z*[*x*], and an error distribution *χ* over the ring *R*_*q*_ = *Z*_*q*_[*x*]/〈*f*(*x*)〉. The parameters *n*, *f*, *q*, and *χ* are public. It assumes that, given the security parameter *k*, there are polynomial-time algorithms that output *f* and *q* and a sample from the error distribution *χ*.

The LE encryption scheme consists of the following three algorithms: KeyGen(·), Enc(·), and Dec(·) (Algorithm 1).

In [24], they apply the techniques of bootstrapping, modulus reduction, and relinearization to realize and to operate on the ciphertexts of the inputs encrypted by multiple, unrelated keys. Therein, they have obtained a secure outsourcing multiparty computation protocol on lattice-based encrypted data under multiple keys of users in one server scenario. However, it is not satisfactory because the interaction in the decryption stage is still inevitable.

In this paper, based on this encryption scheme, we consider the outsourcing problem in two-cloud-servers scenario and succeed to construct a secure noninteractive outsourcing protocol that achieves the least computation and communication complexities for users.

### 3.2. Security Model

In this paper, we will discuss our protocol in the semihonest model and analyze its security using the real-ideal paradigm [5].

Firstly, in the ideal world, the computation of the functionality *F* on users' private inputs is conducted by an additional trusted party that receives *x*_*i*_ from user *P*_*i*_, *i* = 1,2,…, *m*, and returns the result *f*(*x*_1_, *x*_2_,…, *x*_*m*_) to the authorized users *P*_*t*_, while other unauthorized parties do not get any output. Hence, in the ideal world, all users' private inputs are well protected, and only authorized users are able to learn about the result. However, there is no trusted party in the real world, and so all parties have to run a protocol Π to get the desired result. During executing the protocol Π, all parties act semihonestly following the protocol but make effort to gain more information about other parties' inputs, intermediate results, or overall outputs by the transcripts of the protocol. An adversary can corrupt a party to receive all messages directed to it and control the messages to be sent out from it.

Herein, we denote the joint output of the ideal world adversary *S* and the outputs of the remaining parties in an ideal execution for computing the functionality *F* with inputs $\vec{x}=(x_{1},x_{2},\ldots ,x_{m})$ as ${\text{IDEAL}}_{F,S}(\vec{x})$, the joint output of the real world adversary *A*, and the outputs of the remaining parties in an execution of protocol Π with inputs $\vec{x}=(x_{1},x_{2},\ldots ,x_{m})$ as ${\text{REAL}}_{\Pi ,A}(\vec{x})$. Then, we say that protocol Π securely realizes functionality *F* if, for every real adversary *A* corrupting any parties and possibly the cloud servers, there exists an ideal world adversary *S* with black-box access to *A* such that, for all input vectors $\vec{x}$, ${\text{IDEAL}}_{F,S}(\vec{x})\overset{c}{\approx }{\text{REAL}}_{\Pi ,A}(\vec{x})$.

## 4. Our Result

**Figure 1:**
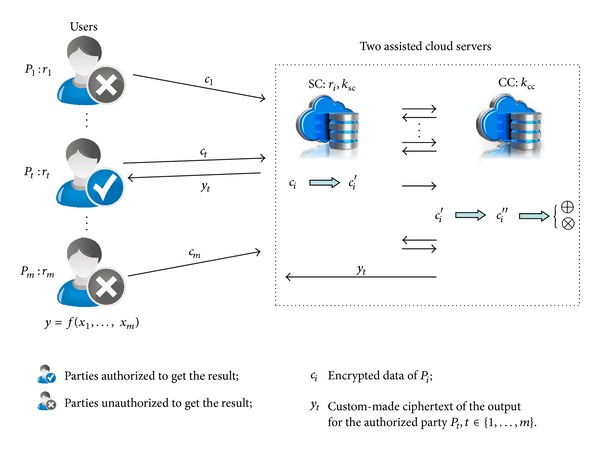
Framework of our construction.

We consider the secure outsourcing computation problem in the multiple users-two-cloud-servers scenario described as follows. There are *m* + 2 parties including *m* users and 2 noncolluding cloud servers: one is called the storing-cloud (SC), and the other is called the computing-cloud (CC). Each user *P*_*i*_ has a private input denoted as *x*_*i*_ and a pair of public-private keys (*pk*_*i*_, *sk*_*i*_) while sharing a private random *r*_*i*_ with SC that has a private number *k*_sc_. CC has a private number *k*_cc_. Users want to outsource the task of computing function *f*(·) on users' private inputs to the two cloud servers. They only provide the ciphertexts of the private data encrypted by a lattice-based encryption scheme under their different public keys and require the cloud servers to give the authorized users the result while keeping the security of the inputs. What is more, users wish that the cloud servers take charge of all of the computations related to the function *f*(·) and that there is noninteraction of users whatsoever so that the computation and communication complexities of each user are independent of the function to be computed. Herein, we deem that the two rounds of inevitable communications and a request from a user to the cloud servers for computing function *f*(·) are the three basic rounds of communication in this paper. Then, for each user, they expect that there are no other interactions at all between any user-to-user or user-to-server except the three basic rounds of communication. Furthermore, the computation complexity of each user depends on the encryption scheme it has used. The framework of our construction can be illustrated in Figure 1.

In this following section, we formally propose our solution denoted as protocol Π for convenience in detail and then analyze its security using the real-ideal paradigm in the semihonest model.

Without loss of generality, we represent the function *f*(·) to be computed by means of arithmetic circuit *C*_*f*_ consisting of any number of addition gates and *l* multiplication gates where each gate has two input wires and one output wire. Then, any functionality can be reduced to the two basic operations, addition and multiplication, over two inputs. Our construction can be summarized in Algorithm 2.

In setup, each user *P*_*i*_ invokes KeyGen(1^*k*^) to compute its public-private keys (*pk*_*i*_, *sk*_*i*_). At the same time, each *P*_*i*_ selects a random *r*_*i*_ and sends it to SC via secure channels, while SC and CC, respectively, choose private numbers *k*_sc_ and *k*_cc_. Assuming that all users' private data *x*_*i*_, *i* = 1,2,…, *m*, are the real inputs of function*f*(·), then each *P*_*i*_ sends *r*_*i*_ · *s*_*i*_ to CC. CC further computes *k*_cc_ · *r*_*i*_ · *s*_*i*_ and sends it to SC. After that, *P*_*i*_ submits the ciphertext *c*_*i*_ of the private input *x*_*i*_ encrypted by its own public key *p*_*i*_ to SC in the upload process.

In computation process, SC firstly midtransforms the outsourced data which are encrypted by different keys of users, and CC further transforms the midtransformed ciphertexts to the ones that are blinded by the same private numbers of the two servers so that CC can operate on the ciphertexts to compute *f*(·). Specifically, for addition/multiplication gate, CC can easily get the result by (*c*_1_^*i*′′^ − *c*_0_^*i*′′^)⊕(*c*_1_^*j*′′^ − *c*_0_^*j*′′^) = *k* · (*x*_*i*_ + *x*_*j*_) and (*c*_1_^*i*′′^ − *c*_0_^*i*′′^)⊗(*c*_1_^*j*′′^ − *c*_0_^*j*′′^) = *k* ^2^ · (*x*_*i*_ × *x*_*j*_). Computing gate by gate following the circuit *C*_*f*_, CC can obtain the intermediate result *y*′ = *k* ^*l*+1^ · *y* = *k*_sc_^*l*+1^ · *k*_cc_^*l*+1^ · *y*.

In order to guarantee that only the authorized user can get the final result, SC and CC cooperatively produce a custom-made result for each authorized user as follows. (Herein, we assume that *P*_*t*_, *t* ∈ {1,2,…, *m*}, is authorized to get the result.) Firstly, CC sends *y*′ to SC. SC removes *k*_sc_^*l*+1^ and adds *r*_*t*_ to compute *y*_*t*_′ = *r*_*t*_ · *k*_cc_^*l*+1^ · *y* and then sends *y*_*t*_′ back to CC. CC finally removes *k*_cc_^*l*+1^ to obtain the custom-made ciphertext *y*_*t*_ = *r*_*t*_ · *y* and sends it to *P*_*t*_.

In the last process, the authorized user *P*_*t*_ obtains the result *y* by removing *r*_*t*_ from *y*_*t*_.

## 5. Analysis

From the protocol described above, the correctness is obvious due to the homomorphic properties of the transformed ciphertexts. We will have a detailed discussion on its security. Note that, before the actual computations which are performed by SC and CC, there are setup and upload processes. We will individually illustrate their security at first. Afterwards, we will prove the security of the core of our protocol, that is, the outsourcing computation process, in the real-ideal framework. Finally, from the composition theorem [5], we can conclude that our protocol is secure.

**Theorem 1**

Protocol Π is secure as long as the LE scheme is secure and SC and CC are noncolluding.

**Proof**

Firstly, we look at the setup and upload processes individually.

In setup, each user, respectively, encrypts its private input by its own public key which is produced by invoking a semantically secure LE scheme. The security of this process is obvious. Afterwards, *P*_*i*_ sends *r*_*i*_ · *s*_*i*_ to CC and CC sends *k*_cc_ · *r*_*i*_ · *s*_*i*_ to SC. Herein, *P*_*i*_'s private key *s*_*i*_ is protected by the blinding factors: *r*_*i*_ which is private to *P*_*i*_ and SC and *k*_cc_ which is private to CC. Therefore, the private keys of users will not be revealed in this process.

In upload, users outsource the encrypted data to SC. Since the LE scheme is semantically secure, given two ciphertexts *c*_*i*_(*m*_1_), *c*_*i*_(*m*_2_) of the two plaintexts *m*_1_, *m*_2_ uploaded by *P*_*i*_, it is computationally infeasible for SC to distinguish the two ciphertexts. Hence, users can store their encrypted data in SC securely.

In outsourcing computation process, SC firstly midtransforms *c*_*i*_ to *c*_*i*_′ and sends *c*_*i*_′ to CC. It is obvious that it is secure since SC blinds the midtransformed ciphertext *c*_*i*_′ by the private number *k*_sc_, which is secret to CC. As to the core of the computation process, we will discuss the security in the real-ideal framework. From the security definition, we say that protocol Π is secure if all adversarial behavior in the real world can be simulated in the ideal model where there exists an additional trusted party to perform all computations related to the function *f*(·) to be computed. We assume that there is a simulator *S* in the ideal world and then prove that it can simulate the semihonest adversary *A* that exists in the real execution. Since CC is able to independently complete addition and multiplication operations, we only need to prove that Add and Mul are secure against the semihonest adversary *A* corrupting CC. We prove this as follows.

Simulator *S*. Run *A* on input{*c*_*S*_(*m*_1_), *c*_*S*_(*m*_2_)}.

Firstly, *S* computes

(1) $$\begin{array}{c} c_{S}\left(m_{1}\right)=\text{Enc}\left(pk_{S},1\right);\\ c_{S}\left(m_{2}\right)=\text{Enc}\left(pk_{S},1\right) \end{array}$$

and sends *c*_*S*_(*m*_1_), *c*_*S*_(*m*_2_) to *A*.

Secondly, *A* sends two ciphertexts *c*_*S*_(*m*_1_*), *c*_*S*_(*m*_2_*) to *S*. Then, *S* computes

(2) $$\begin{array}{c} c_{S}\left(m_{1}^{*}+m_{2}^{*}\right)=c_{S}\left(m_{1}^{*}\right)⊕c_{S}\left(m_{2}^{*}\right);\\ c_{S}\left(m_{1}^{*}\times m_{2}^{*}\right)=c_{S}\left(m_{1}^{*}\right)⊗c_{S}\left(m_{2}^{*}\right) \end{array}$$

and returns  *c*_*S*_(*m*_1_* + *m*_2_*), *c*_*S*_(*m*_1_* × *m*_2_*) to *A*.

Finally, *S* outputs what *A* outputs.

Now, we can prove the security of Add and Mul algorithms by contradiction. Firstly, we assume that the view of the adversary *A* in the real world is distinguishable from the view simulated by the simulator *S*. Then, we could find an algorithm to distinguish the ciphertexts encrypted by the LE encryption scheme, which is contrary to our assumption that the LE is semantically secure. Hence, the view of the adversary *A* in the real world is indistinguishable from the view simulated by the simulator *S*. That is,

(3) $$\begin{array}{c} {\text{IDEAL}}_{F,S}\left(c_{S}\left(m_{i}\right)\right)\overset{c}{\approx }{\text{REAL}}_{\Pi ,A}\left(c_{S}\left(m_{i}\right)\right),\;i=1,2. \end{array}$$

Therefore, the two algorithms Add and Mul are secure. Furthermore, from the composition theorem [5], we can conclude that our protocol is secure as long as the LE scheme is secure and SC and CC are noncolluding in semihonest scenario.

## 6. Conclusion

Only contributing the encrypted forms of their private inputs under their own public keys to gain the desired result of some function on the private inputs via powerful cloud with minimal computations and communications is the optimal method especially when users want to compute some complex function. In this paper, we introduce two noncolluding cloud servers to construct a secure outsourcing multiparty computation protocol on lattice-based encrypted cloud data under multiple keys in semihonest scenario. All computations related to the computing function are in the charge of the two cloud servers. Therefore, the computation complexity of each user only depends on the encryption scheme it has used. What is more, the communication complexity of each user is also independent of the function to be computed and there is no interaction of users whatsoever any more.

## Figures and Tables

**Algorithm 1:**
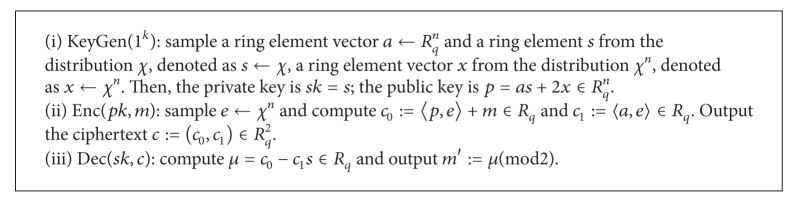


**Algorithm 2:**
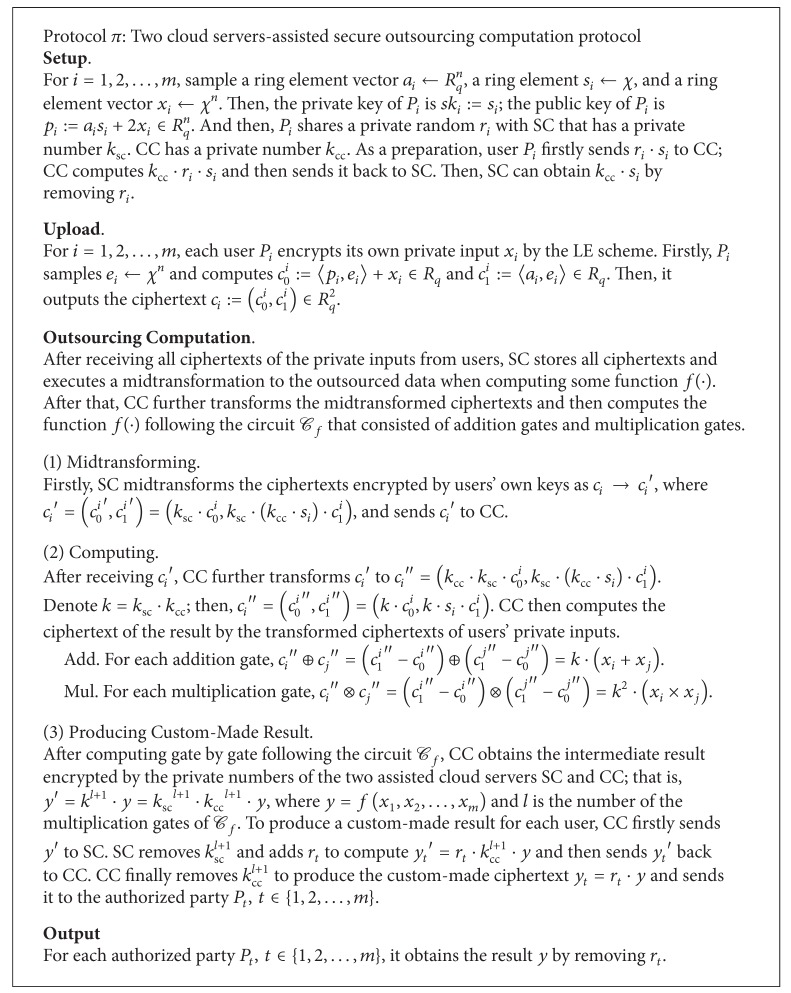


## References

[1] Yao AC Protocols for secure computations.

[2] Lindell Y, Pinkas B (2009). A proof of security of yao’s protocol for two-party computation. *Journal of Cryptology*.

[3] Goldreich OS, Mical S, Wigderson A How to play any mental game.

[4] Goldreich OS Secure multiparty computation.

[5] Goldreich OS (2004). *Foundations of Cryptography: Volume 2, Basic Applications*.

[6] Prabhakaran MM, Sahai A (2013). *Secure Multiparty Computation*.

[7] Fagin R, Naor M, Winkler P (1996). Comparing information without leaking it. *Communications of the ACM*.

[8] Chaum D, Crépeau C, Damgård I Multiparty unconditionally secure protocols.

[9] Damgård I, Pastro V, Smart NP, Zakarias S (2012). Multiparty computation from somewhat homomorphic encryption. *Advances in Cryptology—CRYPTO 2012*.

[10] Lindell Y, Pinkas B (2007). An efficient protocol for secure two-party computation in the presence of malicious adversaries. *Advances in Cryptology—EUROCRYPT 2007*.

[11] Pinkas B, Schneider T, Smart NP, Williams SC (2009). Secure two-party computation is practical. *Advances in Cryptology—ASIACRYPT 2009*.

[12] Armbrust M, Fox A, Griffith R (2010). A view of cloud computing. *Communications of the ACM*.

[13] Velte T, Velte A, Elsenpeter R (2009). *Cloud Computing, a Practical Approach*.

[14] Loftus J, Smart NP (2011). Secure outsourced computation. *Progress in Cryptology—AFRICACRYPT 2011*.

[15] Atallah MJ, Frikken KB Securely outsourcing linear algebra computations.

[16] Kamara S, Mohassel P, Raykova M (2011). Outsourcing multiParty computation. * IACR Cryptology ePrint Archive*.

[17] Peter A, Tews E, Katzenbeisser S (2013). Efficiently outsourcing multiparty computation under multiple keys. * IACR Cryptology ePrint Archive*.

[18] Wang B, Li M, Chow M Computing encrypted cloud data efficiently under multiple keys.

[19] Van Dijk M, Juels A On the impossibility of cryptography alone for privacy-preserving cloud computing.

[20] Gentry C (2009). *A fully homomorphic encryption scheme [Doctoral dissertation]*.

[21] Asharov G, Jain A, López-Alt A, Tromer E, Vaikuntanathan V, Wichs D (2012). Multiparty computation with low communication, computation and interaction via threshold fhe. *Advances in Cryptology—EUROCRYPT 2012*.

[22] Halevi S, Lindell Y, Pinkas B (2011). Secure computation on the web: computing without simultaneous interaction. *Advances in Cryptology—CRYPTO 2011*.

[23] Brakerski Z, Vaikuntanathan V Efficient fully homomorphic encryption from (standard) LWE.

[24] López-Alt A, Tromer E, Vaikuntanathan V (2011). Cloud-assisted multiparty computation from fully homomorphic encryption. *IACR Cryptology ePrint Archive*.

[25] Brakerski Z, Vaikuntanathan V (2011). Fully homomorphic encryption from ring-lwe and security for key dependent messages. *Advances in Cryptology—CRYPTO 2011*.

